# Fabrication of Nanopores Polylactic Acid Microtubes by Core-Sheath Electrospinning for Capillary Vascularization

**DOI:** 10.3390/biomimetics6010015

**Published:** 2021-02-16

**Authors:** Yingge Zhou, Dilshan Sooriyaarachchi, George Z. Tan

**Affiliations:** 1 Systems Science and Industrial Engineering, Binghamton University, Binghamton, NY 13902, USA; yzhou@binghamton.edu; 2 Industrial, Manufacturing & Systems Engineering, Texas Tech University, Lubbock, TX 79409, USA; dilshan.sooriyaarachchi@ttu.edu

**Keywords:** core-sheath electrospinning, nanoporous microtubes, capillary vessels

## Abstract

There has been substantial progress in tissue engineering of biological substitutes for medical applications. One of the major challenges in development of complex tissues is the difficulty of creating vascular networks for engineered constructs. The diameter of current artificial vascular channels is usually at millimeter or submillimeter level, while human capillaries are about 5 to 10 µm in diameter. In this paper, a novel core-sheath electrospinning process was adopted to fabricate nanoporous microtubes to mimic the structure of fenestrated capillary vessels. A mixture of polylactic acid (PLA) and polyethylene glycol (PEO) was used as the sheath solution and PEO was used as the core solution. The microtubes were observed under a scanning electron microscope and the images were analyzed by ImageJ. The diameter of the microtubes ranged from 1–8 microns. The diameter of the nanopores ranged from 100 to 800 nm. The statistical analysis showed that the microtube diameter was significantly influenced by the PEO ratio in the sheath solution, pump rate, and the viscosity gradient between the sheath and the core solution. The electrospun microtubes with nanoscale pores highly resemble human fenestrated capillaries. Therefore, the nanoporous microtubes have great potential to support vascularization in engineered tissues.

## 1. Introduction

Vascularization has been challenging for several decades in the tissue engineering field. The introduction of blood vessels into artificial tissues is one of the most critical steps toward viable organ transplant substitutes. In the last years, remarkable progress was made in the development of bioprinted microchannel networks and decellularized matrices for both artery and vein vascularization [1,2]. However, the finest of the fabricated microchannels are within a scale of several hundred microns in diameter, which is not aligned with the human capillary vessels’ diameter range (5–10 µm) [3,4]. There are three different lining structures for capillary vessels: continuous, fenestrated, and sinusoidal [5,6]. The basement membrane layer and endothelial layer are closed in continuous capillaries, while the endothelial layer is porous in fenestrated and sinusoidal capillaries. The porous structure is believed to improve the efficiency of transportation of biological factors between inside and outside of the capillaries [7]. Similarly, the incorporation of nanoporous microchannels into biomimetic scaffolds can significantly improve the viability of cultured cells inside scaffolds. Therefore, there is a research gap to create biological substitutes for capillary vessels in relevant scale and nanoporous structures.

At centimeter to millimeter scale, electrospun nanofiber mat can be rolled into a tubular structure as a scaffold for engineering vessels [8,9]. However, the diameters of human capillaries are at micron level. To better address the challenge of capillary vascularization in biomimetic scaffolds, numerous efforts have been made. For example, Wong et al. used microcontact imprinting to generate 2D patterns of adhesive proteins on non-cell-adhesive substrates and adhered human umbilical vein endothelial cells (HUVECs) on it. The capillary-patterned cells can migrate and sprout into hydrogels that cover the cells, therefore forming a patterned vasculature formation in hydrogel [10]. Moya et al. combined tissue engineering and microfluidic technology to fabricate a 3D stroma that contains a perfused and interconnected human capillary network [11]. Other advanced manufacturing techniques that were deployed for capillary fabrication include laser-assisted bioprinting [12], electrospinning [13,14,15], and 3D printing [16]. For example, coaxial spinnerets were adopted to fabricate nano-to-micrometer tubes with average tube diameters within 1 µm [17,18]. Polycaprolactone (PCL) is one of the most commonly used materials. The diameter can be modulated by electrospinning process parameters [19]. Studies showed that blending or co-electrospun natural and synthetic polymers could improve the mechanical strength of the fibers and promote vascular formation [20,21,22]. For example, the biocompatibility of the electrospun tubes could be improved by adding collagens into the polymer solutions [18,23,24]. Meanwhile, recent work in bioprinting focused on extruding multi-type materials in a coaxial extrusion system [25,26]. However, the limitation of extrusion nozzle size hinders the formation of microtubules.

Recent research projects have shown the possibility of fabricating 3D aligned nanofibers scaffolds and 2D aligned nanoporous microtubular scaffolds [27,28,29]. The results indicate that the fiber geometry and microtube size can be changed by changing the process parameters and ambient environment settings. Other studies focused on customizing the composition and degree of cross-linking by adding different hydrogels into bioinks [25,30,31]. Overall, there are several drawbacks associated with these fabrication techniques, such as low fabrication efficiency and nonrelevant capillary diameter. Therefore, efficiently fabricated microtubular structures with a diameter of less than 10 µm and surface nanopores remains challenging.

To address the challenge, a novel core-sheath electrospinning strategy has been developed to fabricate polylactic acid (PLA) microtubes with surface nanopores to mimic the fenestrated capillaries. With phase separation and water-soluble polymer core, the porous surface and tubular structure can be obtained [27]. The objectives of this research were to investigate the effects of process parameters, such as flow rate, and material properties, such as solution viscosity on tube diameter and pore size. Our hypothesis was that tubular structure is determined by viscosity ratio of core to the sheath solutions and that nanopores will form on the microtube surfaces due to the rapid evaporation of the solvent. The polymer composition in the sheath solution can also influence the nanopores formation. To test the hypotheses, various combinations of polymer solution flow rate, viscosity levels, as well as different polymer ratios in sheath polymer solution were tested. The results show that the solution viscosity levels, flow rates, and polymer composition in sheath solution all had significant influence on microtube size, and the solution viscosity levels and polymer composition in sheath solution had significant influence on nanopores size. The microtube closure rate was higher when the sheath solution flow rate was high or when there was more polyethylene glycol (PEO) in the sheath solution.

## 2. Materials and Methods

### 2.1. Preparation of Polymer Solutions

Polylactic acid (PLA, M_w_ = 194,000, Ingeo Biopolymer 4032D) pellets were purchased from Jamplast Inc. (Ellisville, MO, USA). Polyethylene glycol (PEO, molecular weight = 300,000) powder and dichloromethane (DCM, ≥99.5%) were purchased from Sigma-Aldrich (St. Louis, MO, USA). Deionized water (DI water) was obtained from PURELAB Classic Water Purification System (ELGA Lab water, High Wycombe, UK).

The 15% and 16% (*w*/*v*) PLA solutions were prepared by dissolving PLA in DCM through magnetic stirring for 4 h at room temperature. Similarly, 3.8% and 4.5% (*w*/*v*) PEO solutions were prepared by dissolving PEO in DCM through magnetic stirring for 4 h at room temperature. The solution ratios were selected based on solubility, viscosity levels, and ease of electrospinning jet formation. Sheath solutions with different PLA and PEO ratios were prepared by adding PEO solution into PLA solution with respective volume ratios. The viscosity of the polymer solutions was measured by a digital rotational viscometer (Brookfield AMETEK, Middleboro, MA, USA).

### 2.2. Electrospinning of Microtubes

The electrospinning process was performed on the TL-Pro-BM robotic electrospinning platform (Tongli Tech, Shenzhen, China) with a 50 kV high voltage power source. A concentric core-sheath spinneret was adopted for this study. The PEO solution and the PLA/PEO solution were extruded from a two-channel syringe pump with independently controlled pump rates. The PLA/PEO solution was delivered to the sheath of the spinneret, and the PEO solution was delivered to the core of the spinneret. The solution and parameter settings are summarized in Table 1. The nozzle size for core and sheath solution were gauge 25 and gauge 18, respectively. The tip-to-ground distance was 150 mm. A positive voltage of 10 kV was applied to the spinneret to induce the electrospinning. The humidity of the chamber was set at 40% by a humidifier. The electrospinning time was set to be 3–5 min.

Based on preliminary data, we hypothesized that the discrepancy of viscosity and flow rate between the core solution and sheath solution would influence the formation of concentric dual-material fibers in electrospinning. We also hypothesized that the morphology of nanopore on the microfibers would change after the water bath treatment by adding water-soluble PEO into PLA as the sheath solution. To investigate the effect of solution viscosity, flow rate, and the ratio of PEO in the sheath solution, we designed a fractional factorial experiment as follows (Table 1). Two levels of viscosity (350 mPa·s and 620 mPa·s), two levels of solution pump rate (1 mL/h and 2 mL/h), and three levels of PLA-to-PEO ratio (by volume) in the sheath solution (10:0, 10:1, and 10:2) were taken. A total of 7 groups of experiments were conducted.

### 2.3. Post-Processing of Microtubes

**Figure 1:**
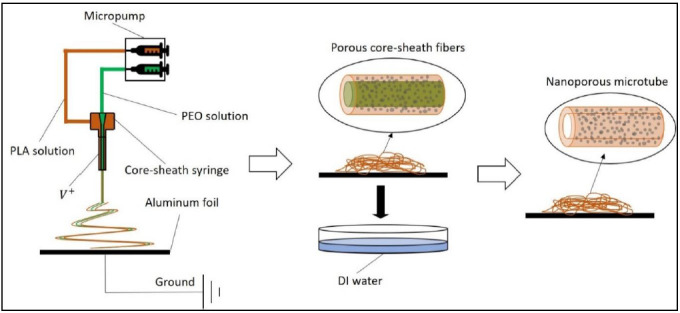
Schematic illustration of the porous microtube fabrication process. PLA = polylactic acid; PEO = polyethylene glycol; DI water = deionized water.

A schematic illustration of processing the electrospun microtubes is shown in Figure 1. By adopting the coaxial spinneret, PLA/PEO and PEO solutions were electrospun simultaneously into microfibers with a core-sheath structure. The porous microfibers were collected by aluminum foil and immersed in deionized water for 2 h to dissolve the PEO. After the water bath, the core was removed and the sheath was thinned, resulting in porous microtubes.

### 2.4. Characterization of Microtubes

To observe the tubular structure, the air-dried PLA microtubes were attached to thin glass slides and immersed in liquid nitrogen for 2 min. The frozen samples were then broken to form cross-sections. The surface and cross-sections of the microtubes were examined under scanning electron microscopy (SEM, Phenom ProX, NanoScience, Alexandria, VA, USA). Fiber diameter and nanopore size were analyzed by ImageJ (National Institutes of Health, Bethesda, MD, USA). The core-sheath concentricity rate was calculated by examining fibers in SEM images. Thirty fibers and 30 pores were selected randomly from each sample.

### 2.5. Cell Attachment

Green fluorescent protein expressing human dermal microvascular endothelial cells (HDMVE, cAP-0005GFP) were purchased from Angioproteomie (Boston, MA, USA). Cells were grown in endothelial growth medium (cAP-02, Angioproteomie) with 0.1% penicillin-streptomycin-amphotericin B solution (ATCC, Manassas, VA, PCS-999-002) until 90% confluency. Electrospun microtubes were cut into 2 × 2 cm and placed in a 6-well culture plate. The microtubes were sterilized by 70% ethanol and rinsed by deionized water for 3 times. Afterward, the microtubes were air dried in the biosafety cabinet with UV light on for 1 h. Cells were detached from the culture flask and resuspended in fresh medium. Approximately 5 × 10^5^ cells were added to the surface of the microtubes and cultured at 37 °C, 5% CO_2_, in an incubator for 7 days. The cell attachment was examined under a fluorescence microscope (EVOS M5000, Thermo Fisher Scientific, Waltham, MA, USA).

## 3. Results

**Figure 2:**
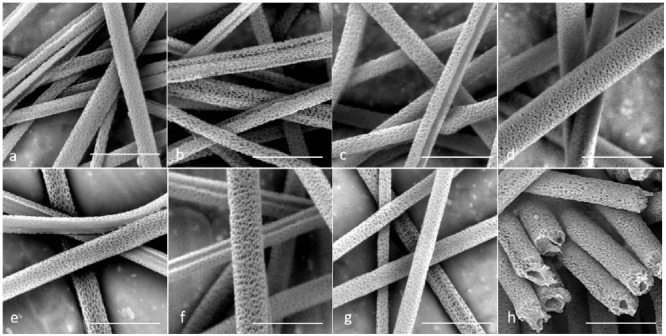
Scanning electron microscopy (SEM) images for all seven groups after water immersion. (**a**–**g**). Groups 1–7; and (**h**) cross-section image of Group 4. Scale bar = 10 µm.

The SEM images of the porous microtubes are shown in Figure 2. Since DCM is highly volatile, the rapid evaporation of DCM during the electrospinning caused a sudden temperature decrease on the surface of the fibers, and the thermodynamic instability induced phase separation of the solution. Meanwhile, the condensation of humidity on the surface of the spinning jets could facilitate nonsolvent-induced phase separation. Both phenomena contributed to the dent formation on the microfiber surface. When the mixture of PLA and PEO was used as the sheath material, a large portion of the dents became pores after the dissolution of PEO. For example, in Group 2 (Figure 2b), the wall of the microtubes was thinned so that there were many penetrating nanopores on the PLA sheath.

The consistency between the core solution and the sheath solution played a critical role in generating a good tubular structure in the core-sheath electrospinning. In Groups 3, 5, and 6, many of the microtubes showed defects or a non-concentric structure. The most common defect was that the sheath failed to fully wrap the core so that core-sheath fibers ended up with a half-round tube structure after the water bath treatment (Figure 2c,e,f). For Group 3, the flow rate of the core solution was higher than that of the sheath solution. The inconsistency in solution flow during the electrospinning caused the structural defect. For Groups 5 and 6, the viscosities of the core and sheath solutions were different. This caused instability of the dual-material jet in the whipping. On the other hand, in Groups 2, 4, and 7, most of the microtubes showed a complete tubular structure without large openings on the wall. In all these groups, the viscosity was kept the same for the core solution and the sheath solution. In Group 4, the flow rate of the sheath solution was higher than that of the core solution, and the electrospun microtubes maintained a good tubular structure. Figure 2h shows the cross-sections of the microtubes in Group 4. It shows that a moderate increase in the flow rate for the sheath solution did not damage the final tubular structure of the microtubes.

**Figure 3:**
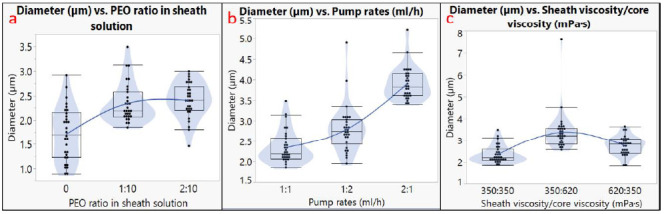
Diameter analysis. Effects of (**a**) PEO ratio in sheath solution; (**b**) pump rates; (**c**) sheath viscosity/core viscosity.

**Figure 4:**
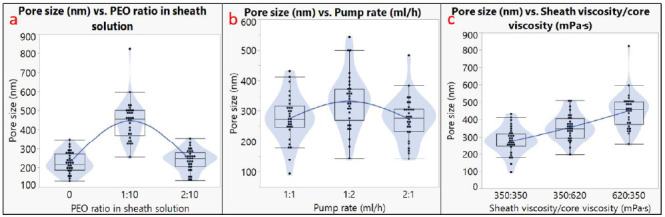
Pore size analysis. Effects of (**a**) PEO ratio in sheath solution; (**b**) pump rates; (**c**) sheath viscosity/core viscosity.

The diameter analysis is shown in Figure 3, and the pore size analysis is shown in Figure 4. The outer diameter of microtubes ranged from 0.9 to 7.6 µm, and the size of the nanopores ranged from 130 to 820 nm. It should be noted that not all the factors had a linear impact on the microtube diameter or the nanopore size. For example, using different pump rates for the sheath and the core solutions generated larger microtubes compared to using the same pump rate; however, a higher pump rate of the sheath solution led to the largest microtube diameter (Figure 3b). The microtube diameter also increased when the viscosity of the core solution increased (Figure 3c). Regarding the nanopore size, the addition of 10% PEO to the PLA sheath solution resulted in a larger pore size, but more PEO (20%) did not increase the pore size (Figure 4a). Having inconsistent viscosities between the core solution and the sheath solution contributed to larger nanopore sizes, and the microtubes showed the largest nanopore size when the sheath solution had a higher viscosity (Figure 4c).

**Figure 5:**
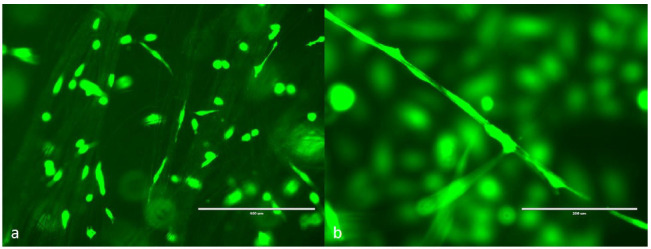
GFP human dermal microvascular endothelial cells (HDMVE) attached on the electrospun microtubes. (**a**) Cells randomly distributed on the microtubes, scale bar = 400 μm; (**b**) cells grown along a single microtube, scale bar = 200 μm.

Endothelial cells were successfully attached to microtubes in 3D space after 24 h. Figure 5 shows the fluorescent cells after 3 days of culturing. Most cells were randomly distributed among the microtubes. Some cells grew along the microtubes to form a continuous line. Because the microtubes were distributed in a 3D space instead of on a 2D surface, some objects were out of focus. Confocal microscopy will be used in the future to capture the 3D images. This preliminary test shows that the electrospun microtubes were compatible with human endothelial cells; therefore, they can be used as scaffolds for capillary vessel engineering.

## 4. Discussion

Electrospinning has been utilized for nanofiber fabrication for many decades. Scaffolds made of micro-nanofibers have shown great potential for tissue engineering. In our previous research projects, a novel divergence electrospinning strategy was developed to scale up traditional 2D nanofiber mats to 3D nanofiber scaffolds with gradient microstructures along the vertical direction [32,33,34,35,36]. The scaffolds’ thicknesses ranged from 2 to 10 cm. Human fibroblasts were cultured in the nanofiber scaffolds and grew along the parallel fibers in 3D space. In this research, nanoporous microtubes were fabricated by core-sheath electrospinning to resemble capillary vessels. The outer diameter of the microtubes ranged from 1 to 8 µm and the average size of the surface nanopores ranged from 100 to 800 nm. The fabrication time of a millimeter-size microtube scaffold in height only took 3–5 min. The incorporation of micro-to-nano fibers into tissue engineered scaffolds has been widely studied in the past decades. For example, Xu et al. prepared poly (lactic-co-glycolic acid) based triblock copolymer microtubes for noninvasive monitoring of bone regeneration [37]. The microtube embedded hydrogel possessed an ideal sustained drug release property. After implanting the composite hydrogel into the tibial defect of rats, the results showed that the hydrogel scaffold was completely degraded after 4 weeks and the tibial defect was repaired after 6 weeks. In another review conducted by Nakielski et al., the challenges and impact of nano- and microfiber morphology on tissue engineering applications such as hemostatic agent was also discussed [38]. It was found that increasing fiber dressing porosity can ease blood absorption and increase clotting factor concentration. Other methods such as surface functionalization can trigger the activation of platelets and lead to faster clot formation. Therefore, our microtubes could be of high potential in the vascularization applications of tissue engineered scaffolds.

In our core-sheath electrospinning process, surface pore formation occurred in the phase separation phenomenon due to the rapid evaporation of the solvent (DCM). DCM has been widely used in the fabrication of porous micro-to-nano fibers using electrospinning. For example, Nguyen et al. obtained porous polycaprolactone (PCL) fibers with DCM and acetone mixture as a solvent for CaP particles coating in bone tissue engineering applications [39]. The polymer-rich phase formed a fibrous matrix, while the mixture solvent-rich phase formed the spherical pores. It was also found that the solvent mixture rate contributed to the formation of the pores. Similarly, Cao et al. fabricated PLA nanoporous fibers by adjusting the composition ratio of DCM and N,N-dimethylformamide (DMF) solvent mixture rate [40]. Natarajan et al. found that higher relative humidity and its miscibility/interaction with DCM solvent might contribute more to the generation of surface porosity [41]. In our study, the viscosity gradient between the core and the sheath solution showed a significant influence on pore size. In addition, the PEO ratio in sheath solution also played an important role in both the wall thickness and the pore size after the water bath treatment. In our experiment, the highest average pore size was obtained when the sheath viscosity was 620 mPa· s and core viscosity was 350 mPa·s. Rezabeigi et al. showed that in the PLA-DCM-hexane electrospinning system, a range of viscosity allowing for the production of porous spherical microfibers exists. Lee et al. also found that in the core-sheath coaxial electrospinning process, the higher viscosity of polymer solutions resulted in higher pore size.

Our diameter analysis showed that the pump rates (solution flow rates), solution viscosity levels, and PEO ratio in the sheath solution had significant influences on the microtube diameter (*p* < 0.05). The PEO ratio in sheath solution and the viscosity difference between the core solution and the sheath solution showed a significant influence on the nanopore size (*p* < 0.05). Both the diameter and the nanopore size increased when the mixture solution of PEO and PLA was used as the sheath solution. Other research projects also showed results that are consistent with our analysis. For example, Duan et al. also found that higher flow rates promoted the formation of larger fibers, since more solution coming out from the nozzle and a larger amount of polymers would form larger fibers [42]. Similarly, Yu et al. suggested that by only increasing the sheath flow rate, the fiber diameter could increase significantly [43]. As for the effect of viscosity, Chen et al. found that higher solution viscosity in core-sheath electrospinning contributes to larger fibers [44]. Given that higher viscosity makes the fiber stream more difficult to be elongated, electrospun fiber is expected to be larger. Additionally, since the diameter was measured by the outer layer of PLA fibers, the effects of sheath solution viscosity would be larger than the core solution. In this study, the wall thickness of the microtubes was not quantitatively analyzed due to the limitation of resources. In the future, fluorescent stain can be added to the sheath solution, and the wall thickness can be measured by confocal microscopy.

In the future, solvent mixtures with other types of solvents, such as dimethylformamide (DMF) [45], will be used in our core-sheath electrospinning process. The ratio of solvent mixtures in both core and sheath solutions will be investigated. The scaffolds can also be incorporated into a hydrogel matrix with endothelial cells for engineering vascularized tissues.

## 5. Conclusions

In this paper, a novel core-sheath electrospinning process was adopted to fabricate nanoporous microtubes for mimicking human fenestrated capillary vessels. The average diameter of these microtubes was on the same scale as the minimum diameter of human capillaries. The results show that the ratio of water to dissolvable polymer in the sheath solution, pump rate, as well as the viscosity gradient between the sheath and the core solution had significant impacts on the microtube diameter. The PEO ratio in the sheath solution and the viscosity gradient substantially influenced the pore size. These nanoporous microtubes can be incorporated into tissue engineering scaffolds to promote angiogenesis and tissue vascularization.

## Figures and Tables

**Table 1:** Materials for the core-sheath electrospinning.

Group Number	Sheath	Core	Viscosity (Sheath) (mPa·s)	Viscosity (Core) (mPa·s)	Flow Rate (Sheath) (mL/h)	Flow Rate (Core) (mL/h)
1	15% PLA	3.8% PEO	350	350	1	1
2	15% PLA/3.8% PEO (10:1)	3.8% PEO	350	350	1	1
3	15% PLA/3.8% PEO (10:1)	3.8% PEO	350	350	1	2
4	15% PLA/3.8% PEO (10:1)	3.8% PEO	350	350	2	1
5	15% PLA/3.8% PEO (10:1)	4.5% PEO	350	620	1	1
6	16% PLA/4.5% PEO (10:1)	3.8% PEO	620	350	1	1
7	15% PLA/3.8% PEO (10:2)	3.8% PEO	350	350	1	1

PLA = polylactic acid; PEO = polyethylene glycol.

## Data Availability

The data presented in this study are available on request from the corresponding author.
